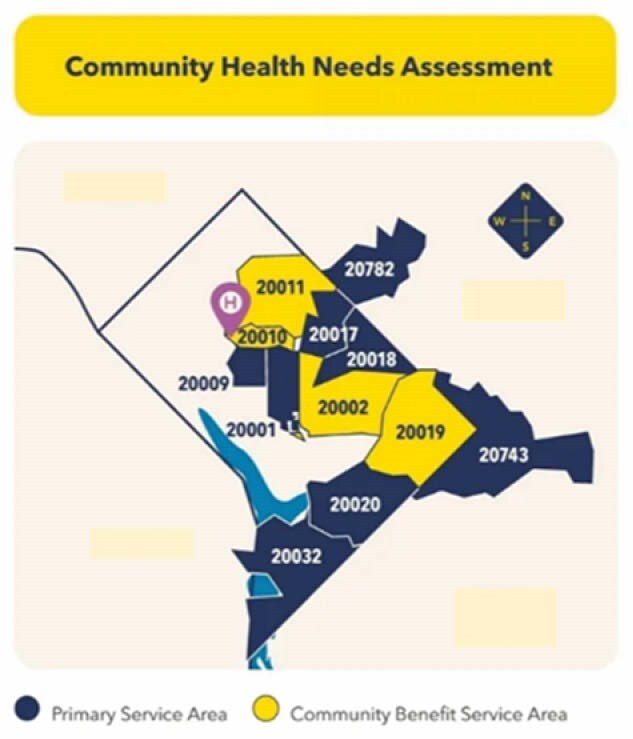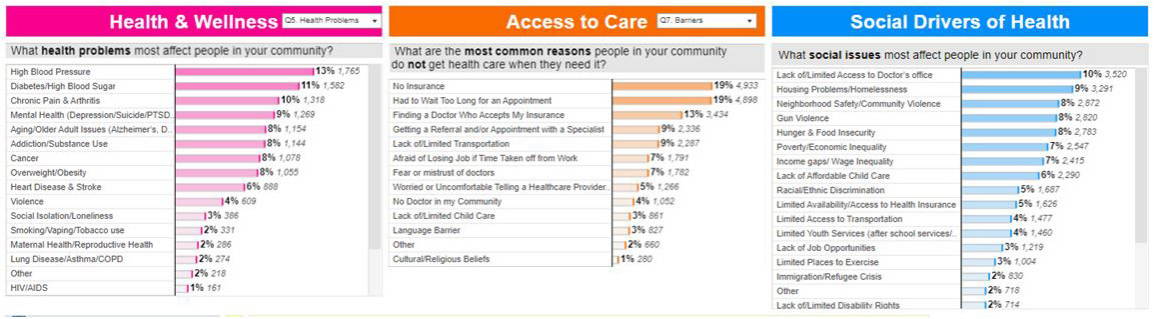# 956 Leveraging Community Health Needs Assessment (CHNA) Data to Inform Burn Outreach and Prevention Strategies

**DOI:** 10.1093/jbcr/iraf019.487

**Published:** 2025-04-01

**Authors:** Angela White, Andrea Miranda

**Affiliations:** MedStar Washington Hospital Burn Center; MedStar Washington Hospital Burn Center

## Abstract

**Introduction:**

Every three years, all not-for-profit healthcare systems conduct a Community Health Needs Assessment (CHNA) to identify health disparities. The current CHNA for the institution serving as home to a regional burn center in a major metropolitan area was carried out from September 2023 through October 2023. The assessment is used to evaluate health challenges in catchment areas facing different populations. The goal of this project was to leverage this database to assess the needs of the population serviced by this regional burn center to potentially better target burn outreach focus areas.

**Methods:**

The CHNA process involved a multifaceted approach to data collection and analysis, ensuring that a wide range of perspective and insights were considered. The CHNA incorporated various data sources, including quantitative secondary population level data, hospital healthcare utilization data, a community survey, and qualitative community input sessions. The CHNA zip codes most commonly serviced by our regional burn center were queried to identify the top health problems and issues identified by the community. The three focus areas, Health and Wellness (N = 13865), Access to Care (N= 26,407), and Social Drivers of Health (N= 35,796) reported responses to the following survey questions, which were the focus of the present data collection to leverage for burn outreach opportunities: What are the health problems that affect people in your community?; and What are the most important issues that affect quality of life in your community?

**Results:**

In the Health and Wellness category, the top health problems or most important issues were: Addiction/substance abuse = 8% (1144), Diabetes/High blood pressure = 11% (1582), Mental Health (Depression/suicide/PTSD/trauma = 9% (1269), Aging/older adult issues (Alzheimer’s, Dementia, Falls) = 8% (1154), Smoking/vaping/tobacco use = 2% (331). In the Access to Care category, Limited access to transportation = 9% (2287) was the top response. For Social Drivers of Health, the top responses were Limited Availability/ access to Doctor’s office = 10% (3520), Housing problems/ homelessness =9% (3,291), Poverty = 7% (2547), Limited access to education = 1% (429). These data were collected and added to existing burn outreach planning tools used by our center to consider future areas of focus.

**Conclusions:**

The findings indicated a need to focus burn prevention efforts in the following areas: senior outreach, mental health, chronic disease, transportation, social services, and substance abuse.

**Applicability of Research to Practice:**

Key barriers were identified through the CHNA surveys. The findings highlight the need for tailored implementation strategies. The CHNA can target existing areas of increased burn injuries with program-specific metrics. Outreach strategies include identifying factors that increase the risk of burn injuries and knowing where to be more accessible for prevention efforts.

**Funding for the Study:**

N/A